# Impact of bacterial exposure on the mechanical behavior of nickel–titanium open-coil springs

**DOI:** 10.2340/biid.v13.46766

**Published:** 2026-09-18

**Authors:** Diego Paul Hidalgo Muñóz, Mauricio Aguirre Balseca, Marjory Elizabeth Vaca Zapata, Karina Maria Salvatore Freitas, Stalin Wladimir Tamami Tualombo

**Affiliations:** a Department of Health Sciences, University of the Hemispheres, Quito, Ecuador; b Department of Orthodontics, Ingá University Center UNINGA, Maringá, Brazil

**Keywords:** nickel–titanium springs, force alteration, *Streptococcus mutans*, orthodontics, corrosion

## Abstract

**Objective:**

This *in vitro* study aimed to evaluate the effect of *Streptococcus mutans* on the force alteration of nickel–titanium (NiTi) open-coil springs under constant compression.

**Materials and methods:**

Sixty NiTi open-coil springs from three commercial brands (3M, Ormco, and American Orthodontics [AO]) were standardized to a resting length of 15 mm and compressed with a 240-g load using an Instron^®^ universal testing machine. Each brand was divided into control (immersed in artificial saliva) and experimental (exposed to *S. mutans* in Brain Heart Infusion medium) conditions. Samples were incubated at 37°C for 30 days, after which force levels at 0.5, 3.25, and 6.5 mm displacements were remeasured. Data were analyzed with paired *t*-tests, one-way analysis of variance, and Tukey’s *post hoc* tests (*p* < 0.05).

**Results:**

All brands exhibited force increases after 30 days, though with heterogeneous patterns. 3M springs maintained relatively stable behavior, with consistent force increments across all displacements in both conditions. AO springs showed irregular responses, with losses at some displacements and substantial gains at others, especially under bacterial exposure. Ormco springs displayed the highest variability and most pronounced increases, with force gains exceeding 80 gf, suggesting possible microstructural modifications.

**Conclusions:**

Force stability in NiTi open-coil springs is brand-dependent and influenced by bacterial exposure. While 3M springs demonstrated predictable performance, AO and Ormco exhibited less consistent behavior, which may risk excessive force application during clinical use. These findings highlight the importance of considering biological and environmental factors in the selection and monitoring of NiTi coil springs.

## Introduction

Nickel–titanium (NiTi) alloys revolutionized orthodontics with their unique properties of superelasticity and shape memory, enabling the application of light and continuous forces over extended periods. Initially introduced as stabilized martensitic wires in the 1970s, their clinical versatility expanded with the discovery of stress-induced martensitic transformation, which allowed predictable tooth movement with minimal side effects such as root resorption or pain [1, 2]. Beyond archwires, NiTi has also been employed in the manufacture of coil springs, which have become essential auxiliaries in space opening, molar distalization, and anchorage reinforcement. Open-coil springs, in particular, are favored because they generate relatively constant expansive forces compared with stainless steel or elastomeric alternatives [3, 4].

Despite these advantages, clinical evidence and *in vitro* studies have shown that NiTi coil springs are subject to progressive force degradation when compressed over time, with reports of up to 40% loss within the first weeks of activation [5, 6]. The extent of this degradation depends not only on the spring design, degree of compression, and manufacturing quality but also on environmental conditions, which makes their actual performance *in vivo* difficult to predict [7, 8]. This variability represents a critical limitation, as the efficiency of orthodontic treatment relies on the ability of the appliance to deliver predictable biomechanical forces.

The oral cavity provides a particularly complex and dynamic environment. Temperature fluctuations, salivary composition, pH variation, and, most importantly, bacterial colonization contribute to alterations in the structural integrity and mechanical properties of orthodontic alloys [9, 10]. Among the diverse microbial species inhabiting the oral cavity, *Streptococcus mutans* is of particular concern due to its acidogenic and aciduric properties, its central role in the initiation of dental caries, and its ability to form resilient biofilms on orthodontic surfaces [11, 12]. Evidence suggests that *S. mutans* not only increases the risk of enamel demineralization but also accelerates corrosion and surface degradation of metallic orthodontic devices, including NiTi alloys [9, 10, 13].

Recent experimental studies have shown that *S. mutans* colonization of NiTi alloys can significantly alter their corrosion behavior and contribute to the decline of mechanical properties [9, 12]. In addition, NiTi open-coil springs have been demonstrated to lose force more rapidly in artificial saliva than in dry conditions [14], and environmental variations, including temperature extremes, also exacerbate force degradation [3]. These findings underscore the plausibility that *S. mutans* may play a direct role in accelerating force decay in NiTi open-coil springs under constant compression, a topic that remains underexplored.

The interaction between bacterial biofilm and NiTi surfaces has been shown to reduce fatigue resistance, modify surface morphology, and impair the long-term performance of orthodontic appliances [9, 10]. However, most available studies have investigated archwires rather than coil springs, leaving limited data regarding how *S. mutans* specifically influences the force decay of NiTi open-coil springs subjected to constant compression. Given that open-coil springs are widely used in clinical orthodontics for space management and anchorage control, understanding this interaction is highly relevant.

In light of these considerations, the present *in vitro* experimental study was designed to evaluate the effect of *S. mutans* on the force alteration of NiTi open-coil springs maintained under constant compression in controlled conditions. By comparing specimens exposed to artificial saliva with and without *S. mutans*, this research seeks to provide new insights into the biological and mechanical challenges faced by NiTi coil springs in the oral environment and to contribute to the development of strategies aimed at enhancing their clinical performance and durability.

## Material and methods

### Study design

This investigation was designed as an experimental *in vitro* study aimed at evaluating the effect of *S. mutans* on the force alteration of NiTi open-coil springs subjected to constant compression.

### Sample selection

A total of 60 NiTi open-coil springs from three different commercial brands were selected by non-probabilistic convenience sampling.

#### Inclusion criteria

- Springs without visible defects or manufacturing anomalies.
- Springs not previously used or subjected to mechanical stress.

#### Exclusion criteria

- Springs with deformities, fractures, or irregularities detected during the initial inspection.
- Springs showing alterations derived from cutting or handling procedures.

#### Standardization of samples

**Figure 1:**
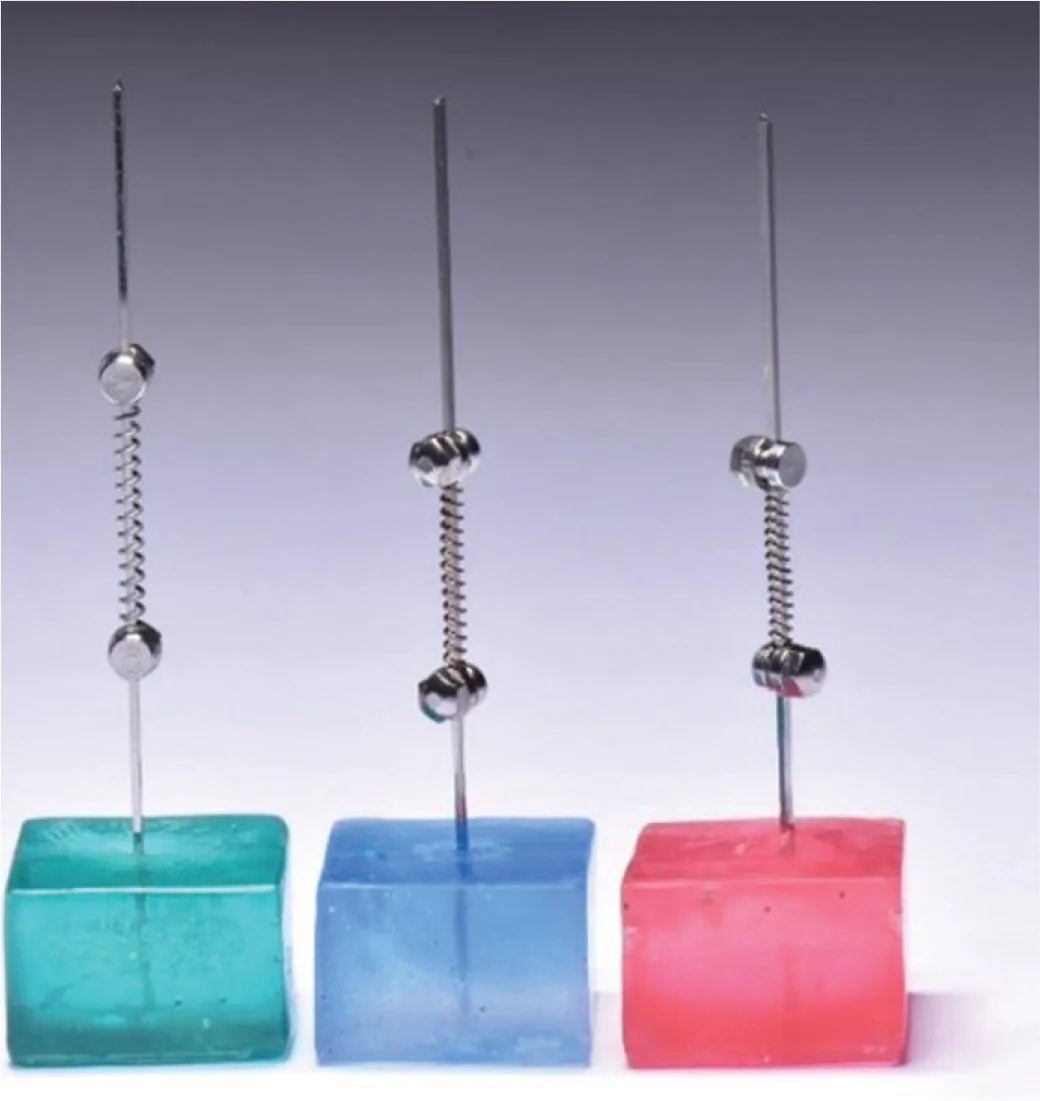
Acrylic cubes with springs prior to compression.

All NiTi springs were standardized to a resting (unloaded) length of 15 mm, that is, the original free length of the spring before compression (Figure 1). Sectioning was performed using orthodontic pliers, and the resting lengths were verified with a digital caliper. The springs were subsequently compressed under a constant 240-g load, and force values were recorded at displacements of 0.5, 3.25, and 6.5 mm relative to this resting length.

**Figure 2:**
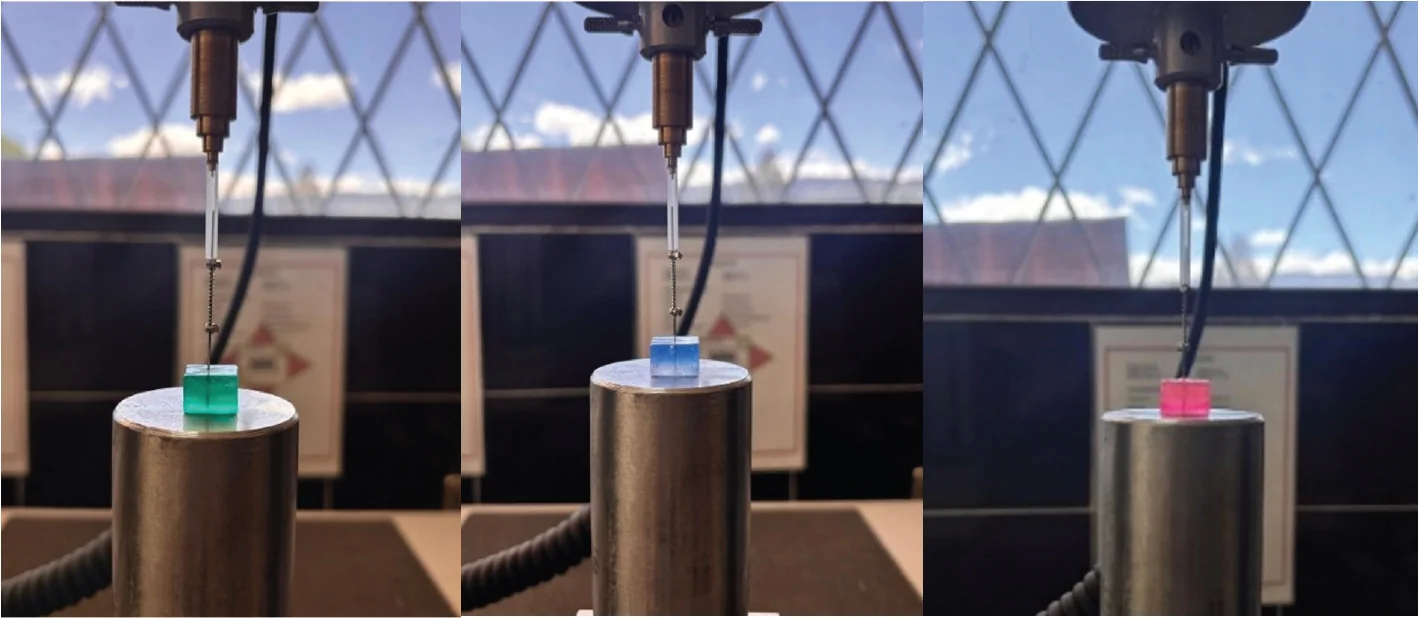
Springs compressed with an Instron machine.

### Brands and experimental conditions

The 60 springs were distributed according to the commercial brand, 3M, Ormco, and American Orthodontics (AO), with 20 springs per brand. Within each brand, the springs were randomly assigned to one of two conditions (*n* = 10 each): a control condition, in which springs were immersed in artificial saliva, and an experimental condition, in which springs were exposed to *Streptococcus mutans* in Brain Heart Infusion (BHI) broth.

### Force application and monitoring

Compression was performed using an Instron universal testing machine, which ensured the application of a standardized 240-g load. The device also provided digital records of displacement, time, and exact force values applied, guaranteeing reproducibility of the experimental conditions.

### Immersion conditions

- Control subgroups (*n* = 10 per brand): springs immersed in sterile glass flasks containing artificial saliva (Saliv^®^), fully covering the samples.
- Experimental subgroups (*n* = 10 per brand): springs immersed in sterile flasks containing BHI broth inoculated with *S. mutans*.

All containers were hermetically sealed and incubated at 37°C for 30 days, with daily monitoring and recording of temperature to avoid deviations that could compromise experimental reliability.

### Retrieval and preparation of samples

After 30 days, under sterile conditions in a laminar flow hood, the immersion solutions were discarded. Springs were removed with sterile tweezers, placed on autoclaved absorbent paper to remove excess liquid, and air-dried within the laminar flow hood.

Once completely dried, all springs were stored in sterile, labeled containers until subsequent mechanical testing and analysis.

### Statistical analysis

The data were organized in Microsoft Excel and analyzed using IBM SPSS Statistics version 25.0 (IBM Corp., Armonk, NY, USA) and Python (SciPy library). Descriptive statistics were expressed as mean ± standard deviation (SD). The Shapiro-Wilk test was applied to verify the normality of the data, and Levene’s test was used to evaluate homogeneity of variances.

For within-brand comparisons (before vs. after 30 days of immersion), dependent *t*-tests were applied.

For between-brand comparisons (among the three brands before and after 30 days, within the same condition), one-way analysis of variance (ANOVA) was performed when assumptions of normality and homogeneity were met, followed by Tukey’s *post hoc* test for multiple comparisons. In cases of non-normal distribution, the Kruskal-Wallis test with the Dunn–Bonferroni correction was applied.

All tests were two-tailed, and the significance level was set at *p* < 0.05.

## Results

A total of 60 NiTi open-coil springs from three commercial brands (3M, AO, and Ormco) were evaluated for the force generated at displacements of 0.5, 3.25, and 6.5 mm, both at baseline (T0) and after 30 days (T30), under control and experimental conditions.

### Baseline force (T0)

At baseline, the mean force ranged from approximately 26–53 gf at 0.5 mm, 155–181 gf at 3.25 mm, and 150–239 gf at 6.5 mm across the three brands. Values for each brand and displacement are presented in Table 1.

**Table 1:** Mean displacement (mm) and force (gf) generated by the NiTi open-coil springs of all brands at baseline (T0), under control and experimental conditions, at displacements of 0.5, 3.25, and 6.5 mm.

Range		0.5 mm		3.25 mm		6.5 mm	
Brands	Group	Displacement (mm) Mean	Force (gf) Mean ± SD	Displacement (mm) Mean	Force (gf) Mean ± SD	Displacement (mm) Mean	Force (gf) Mean ± SD
3M	Control	0.55	26.36 ± 7.64	3.24	154.66 ± 12.84	6.49	238.77 ± 5.22
	Exp.	0.55	29.69 ± 10.99	3.24	156.45 ± 13.73	6.49	243.70 ± 3.02
AO	Control	0.55	36.01 ± 5.86	3.25	180.56 ± 22.52	6.49	174.99 ± 1.17
	Exp.	0.55	35.37 ± 7.42	3.25	174.99 ± 18.37	6.50	241.39 ± 2.07
Ormco	Control	0.55	52.85 ± 33.45	3.245	160.99 ± 31.29	6.14	150.00 ± 1.97
	Exp.	0.55	53.17 ±16.62	3.25	150.00 ± 19.48	6.49	240.00 ± 1.82

Values are expressed as means. 3M, AO, and Ormco denote the three commercial brands (AO, American Orthodontics). Control, springs immersed in artificial saliva; Experimental (Exp.), springs exposed to *Streptococcus mutans* in Brain Heart Infusion broth. gf: grams-force; SD: standard deviation.

### Force after 30 days (T30)

After 30 days, the mean force recorded under the control and experimental conditions ranged from approximately 31–77 gf at 0.5 mm, 159–235 gf at 3.25 mm, and 210–272 gf at 6.5 mm. Values for each brand, displacement, and condition are presented in Table 2.

**Table 2:** Mean displacement (mm) and force (gf) generated by the NiTi open-coil springs of all brands after 30 days (T30), under control and experimental conditions, at displacements of 0.5, 3.25, and 6.5 mm.

Range		0.5 mm		3.25 mm		6.5 mm	
Brands	Group	Displacement (mm) Mean	Force (gf) Mean ± SD	Displacement (mm) Mean	Force (gf) Mean ± SD	Displacement (mm) Mean	Force (gf) Mean ± SD
3M	Control	0.55	38.93 ± 5.93	3.28	162.23 ± 4.85	6.53	264.80 ± 2.28
	Exp.	0.54	31.06 ± 4.73	3.259	159.15 ± 10.82	6.53	267.78 ± 2.02
AO	Control	0.53	38.01 ± 2.99	3.253	168.09 ± 7.53	6.54	210.22 ± 1.16
	Exp.	0.55	52.83 ± 11.57	3.25	210.22 ± 8.47	6.54	264.26 ± 2.28
Ormco	Control	0.53	77.34 ± 15.01	3.25	225.32 ± 12.79	6.20	235.06 ± 4.87
	Exp.	0.54	56.91 ± 4.06	3.24	235.06 ± 3.49	6.49	271.59 ± 2.32

Values are expressed as means. 3M, AO, and Ormco denote the three commercial brands (AO, American Orthodontics). Control, springs immersed in artificial saliva; Experimental (Exp.), springs exposed to Streptococcus mutans in Brain Heart Infusion broth. gf: grams-force; SD: standard deviation.

### Force variation (ΔF)

All three brands showed a positive force variation after 30 days (ΔF > 0; Table 2), statistically significant by paired *t*-tests (*p* < 0.05). One-way ANOVA showed a statistically significant difference in ΔF among brands (*F* = 17393.6; *p* < 0.0001; Table 3). Tukey *post hoc* testing confirmed significant differences in all pairwise comparisons (*p* < 0.001; Table 4): ΔF was significantly greater for Ormco than for AO and 3M, and significantly greater for AO than for 3M.

**Table 3:** One-way ANOVA for the force variation (ΔF) among the three commercial brands.

Source	Sum of squares	Degrees of freedom	Mean square	*F*	*p*
Group	10353.013	2	5176.507	17393.6	< 0.001*
Residual	8.955	27	0.332	-	-

ΔF, difference between the force at 30 days and at baseline (T30 − T0); Group, between-brands variation; Residual, within-group (error) variation; F: F statistic; p: probability value; ANOVA: analysis of variance.

* Statistically significant (*p* < 0.05).

**Table 4:** Tukey post hoc pairwise comparisons of the force variation (ΔF) among the three commercial brands.

Brand 1	Brand 2	Mean difference	*p*	Lower	Upper	Reject H₀
3M	AO	-4.25	0.001*	-5.20	-3.30	Yes
3M	Ormco	-37.84	0.001*	-38.79	-36.89	Yes
AO	Ormco	-33.59	0.001*	-34.54	-32.64	Yes

Mean difference, difference in the mean ΔF between Brand 1 and Brand 2 (gf); Lower and upper, lower and upper limits of the 95% confidence interval; Reject H₀, rejection of the null hypothesis of equal means. 3M, AO (American Orthodontics), and Ormco denote the commercial brands.

* Statistically significant (*p* < 0.05).

## Discussion

The findings of this study demonstrate that NiTi open-coil springs, when immersed for 30 days in artificial saliva and exposed to *S. mutans*, exhibited heterogeneous behavior in terms of force generation. A comparison of the initial (Table 1) and post-exposure forces (Table 2) revealed that all three commercial brands tested showed overall increases in exerted forces, although the magnitude and pattern of these changes differed considerably.

For the 3M springs, both the control and experimental conditions produced consistent force increments across all displacements, with comparable magnitudes under the two conditions (Table 2). These results suggest that 3M springs maintained favorable mechanical stability over prolonged simulated clinical exposure.

The AO springs displayed a less consistent profile. Under the control conditions, a slight gain at the smallest displacement was followed by a reduction at the intermediate displacement, whereas the experimental condition showed more substantial gains, particularly at the intermediate displacement (Table 2). This discrepancy may be related to delayed fatigue phenomena or elastic reactivation influenced by acidic by-products of *S. mutans* metabolism [9, 10].

Ormco springs exhibited the highest variability and the most pronounced changes among the three brands (Table 2). Such alterations could indicate structural changes in the NiTi alloy, potentially driven by surface oxidation or phase-transformation dynamics that enhance force expression rather than reduce it [1, 15].

These results diverge from those of Prado et al. [16], who reported significant force loss in NiTi open-coil springs after prolonged immersion in humid media, particularly in Orthopli™ springs, which showed up to 30% reduction. A key methodological distinction is that the present study incorporated *S. mutans* exposure, a biological factor absent in Prado’s protocol. This microbial challenge may induce alloy modification, through acidic corrosion or altered austenite–martensite balance, that triggers adaptive mechanical responses. Our findings therefore highlight the importance of incorporating biological variables into *in vitro* models to better simulate intraoral conditions.

Consistent with prior reports, bacterial biofilm interaction can accelerate surface degradation and modify mechanical properties of NiTi alloys [9, 10]. Beyond the cariogenic potential of *S. mutans*, studies have highlighted that acidic environments and bacterial by-products can directly influence phase transformations and corrosion of NiTi alloys. Shen et al. [17] reported that biofilm-related acidity alters surface morphology and reduces fatigue resistance in NiTi instruments, which can be extrapolated to orthodontic coil springs under prolonged intraoral exposure. Similarly, Ren et al. [18] described how bacterial biofilms modulate metal surface interactions, providing additional evidence that microbiological factors may exacerbate material degradation in complex ecological niches such as the oral cavity. However, our results suggest that such interactions do not uniformly reduce force; in some cases, they may enhance spring stiffness depending on brand-specific microstructure and manufacturing processes.

Brauchli et al. [19] demonstrated linear force–deflection behavior in 23 NiTi open-coil springs, establishing baseline expectations for mechanical response. The present results show that this linearity may be altered after 30 days of microbial and chemical exposure, underlining the need to assess long-term stability beyond initial mechanical properties. Similarly, Lubinsky [20] documented reductions in deactivation force with increased activation, particularly in AO springs. Our study expands this perspective, revealing that both activation level and prolonged biological exposure contribute to variability in force retention.

Environmental factors such as temperature fluctuations have also been shown to influence NiTi force stability [3]. Similarly, Parvizi and Rock [21] observed that thermal fluctuations significantly modify the load/deflection characteristics of NiTi wires, reinforcing the importance of considering intraoral temperature changes in the long-term stability of open-coil springs. Walker et al. [22] also demonstrated that exposure to prophylactic agents, particularly fluoride solutions, can further accelerate force degradation and corrosion in NiTi alloys, which may occur in parallel with bacterial and pH-mediated effects in the oral cavity. These findings align with the view that multiple intraoral variables, such as temperature, pH, and biofilm, must be considered simultaneously to approximate clinical performance.

From a clinical standpoint, these outcomes carry significant relevance for orthodontic treatment planning. The pronounced increases in Ormco and AO springs could inadvertently generate excessive forces, risking undesirable effects on periodontal tissues and undermining controlled tooth movement. Conversely, the mechanical stability demonstrated by 3M springs suggests greater predictability, potentially reducing the risk of overloading and improving patient comfort.

These results emphasize the need for clinicians to critically evaluate spring performance beyond manufacturer specifications, incorporating evidence from *in vitro* and *in vivo* studies. Advances such as antimicrobial surface coatings, hybrid alloy formulations, and nanotechnology-based modifications [23, 24] hold promise for improving both biomechanical stability and resistance to microbial colonization.

Despite providing novel insights, this study is limited by its *in vitro* design and relatively short evaluation period. Importantly, biofilm formation on the springs was not directly confirmed: no scanning electron microscopy (SEM) or colony-forming unit (CFU) counts were performed to verify effective colonization by *S. mutans*. The changes observed in the experimental subgroups therefore reflect exposure to a *S. mutans* culture rather than a confirmed biofilm-mediated effect. The intraoral environment involves complex interactions among temperature changes, salivary proteins, dietary factors, and multispecies biofilms that were not fully replicated here. Future research should incorporate longer observation periods, microstructural analyses, and diverse bacterial species to clarify the mechanisms underlying force alterations. Additionally, exploring combined approaches, such as antimicrobial coatings coupled with thermal treatments, may provide clinically relevant solutions to enhance spring reliability.

## Conclusions

This study demonstrates that force stability in NiTi open-coil springs is not uniform and varies significantly across commercial brands. While 3M springs maintained relatively stable and predictable performance, AO and Ormco springs exhibited considerable variability, in some cases, showing force increases after prolonged exposure to artificial saliva and *S. mutans*.

The differential responses suggest that composition, thermal treatment, and microstructural features critically determine spring performance. Clinically, these findings underscore the importance of selecting springs not solely based on manufacturer data, but also on evidence of their behavior under simulated biological conditions.

In practical terms, uncontrolled force gains may compromise treatment precision and periodontal health, warranting careful monitoring when using brands with less predictable behavior. Finally, given the scarcity of data on biological influences on NiTi springs, further research is essential to validate these findings and guide the development of next-generation materials with enhanced biomechanical stability and antimicrobial resistance.

## Data Availability

The data generated and analyzed during the current study are available from the corresponding author on reasonable request.
